# Surgical treatment of a solitary pulmonary metastasis from eyelid sebaceous carcinoma: report of a case

**DOI:** 10.1186/1477-7819-12-108

**Published:** 2014-04-23

**Authors:** Kaoru Kaseda, Takashi Ohtsuka, Yuichiro Hayashi, Katsura Emoto, Keisuke Asakura, Ikuo Kamiyama, Taichiro Goto, Mitsutomo Kohno

**Affiliations:** 1 Department of Surgery, Section of General Thoracic Surgery, Keio University, 35 Shinanomachi, Shinjuku-ku, Tokyo 160-8582, Japan; 2 Department of Pathology, School of Medicine, Keio University, Tokyo, Japan

**Keywords:** Sebaceous carcinoma, Lung metastasis, Solitary metastasis

## Abstract

**Background:**

Ocular sebaceous carcinoma is an uncommon, aggressive ocular neoplasm with potential for regional and distant metastasis.

**Case presentation:**

A 77-year-old woman was found to have a solitary pulmonary lesion 6 years after the initial treatment of sebaceous carcinoma of the eyelid. Video-assisted lung wedge resection of an undetermined pulmonary nodule was carried out successfully. Microscopically, the tumor showed foamy cytoplasm and atypical nuclei, consistent with metastasis of eyelid sebaceous carcinoma.

**Conclusion:**

This is the first case report of resected solitary pulmonary metastasis of eyelid sebaceous carcinoma. Pulmonary resection is a good option for the treatment and diagnosis of metastatic eyelid sebaceous carcinoma.

## Background

Sebaceous carcinoma of the eyelid is a relatively rare malignant tumor, and accounts for less than 1% of all eyelid tumors [1]. As well as being a rare tumor, sebaceous carcinoma can mimic other benign inflammatory and malignant processes, thus errors or delays in diagnosis are not unusual [2-5]. Although local management strategies for this tumor have previously been described [6-10], very few reports have focused on the patterns of metastasis of this tumor and the treatment strategies for such metastases [7,8]. Here, we report a case of solitary lung metastasis of eyelid sebaceous carcinoma, and discuss the clinical implication of surgery for a solitary pulmonary metastasis from sebaceous carcinoma.

## Case presentation

**Figure 1:**
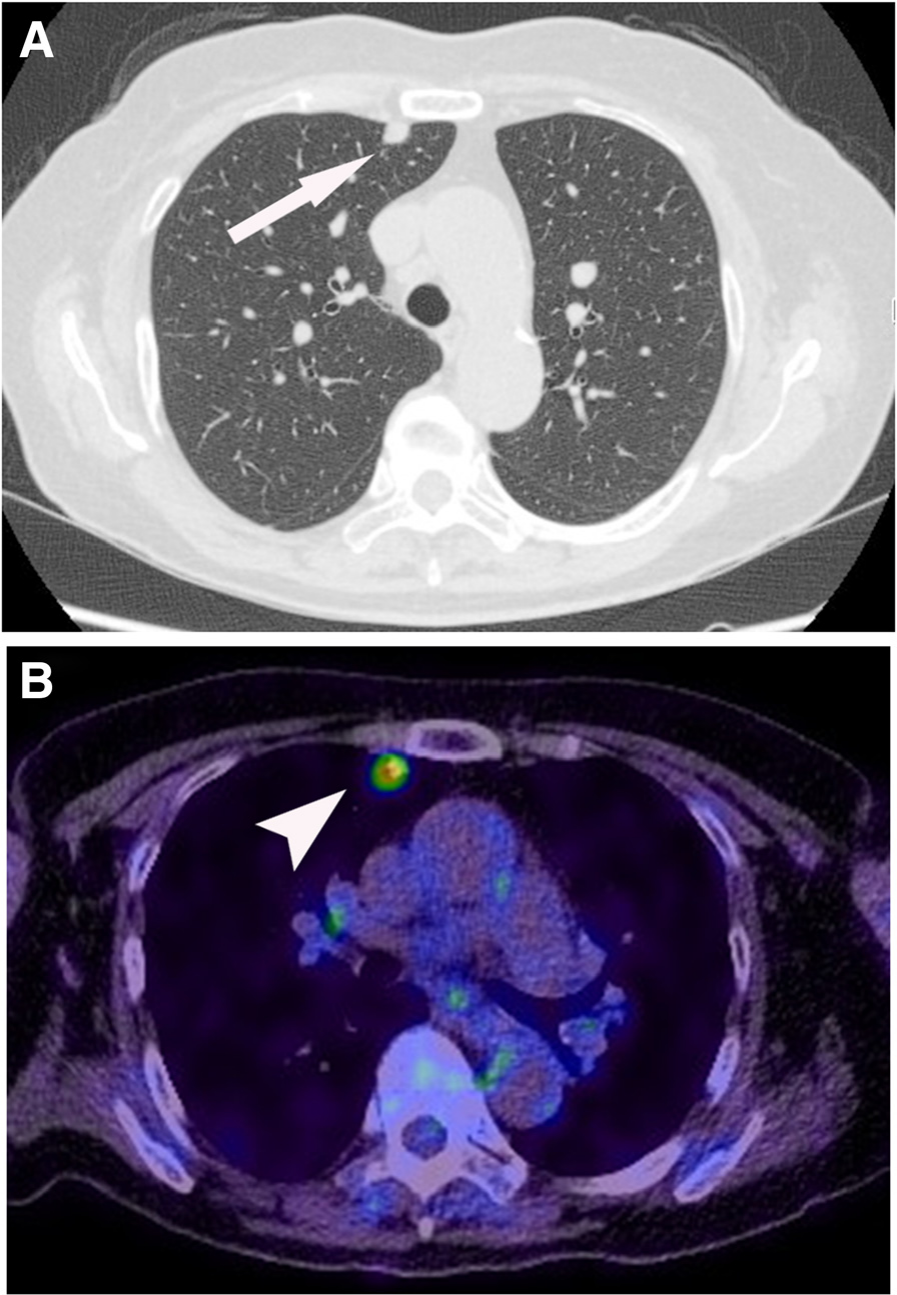
**Computed tomography (CT) and positron emission tomography of the tumors. (A)** Chest CT showed a 1.1 cm nodule in the anterior segment of the right upper lobe (arrow). **(B)** PET-CT showed fluorodeoxyglucose accumulation with a Standardized uptake value (SUV) of 3.7 (arrowhead).

**Figure 2:**
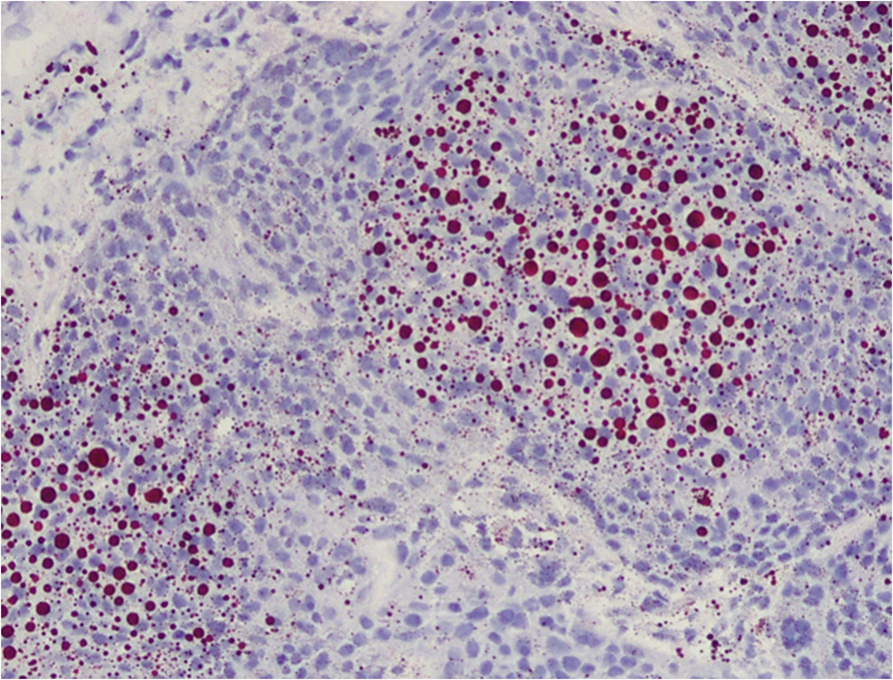
**Accentuation of lipid by staining.** The lipid globules have a red color (frozen sections, oil red O, magnification × 100).

**Figure 3:**
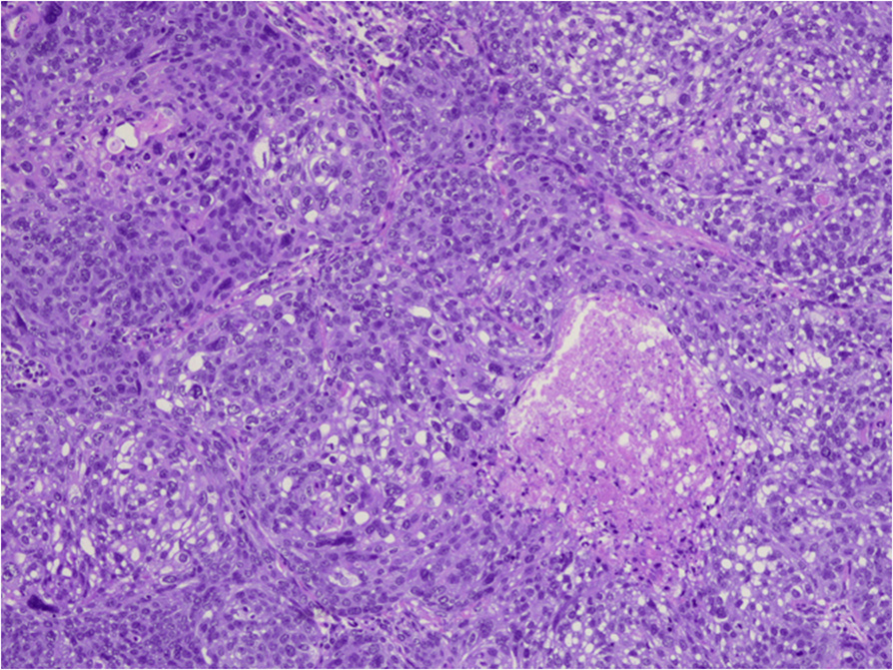
**Sebaceous carcinoma cells.** Foamy and frothy cytoplasm and atypical nuclei, occurred with numerous lipid globules within the cytoplasm of the tumors cells, seen as clear spaces (hematoxylin and eosin, magnification × 100).

A 77-year-old woman underwent left upper lid resection in April 2006 for sebaceous carcinoma of the eyelid. The surgical margin was negative for cancer cells. In January 2008, she had developed a recurrence in the left upper eyelid, and underwent radiotherapy with a total dose of 57.6 Gy of proton beam therapy followed by orbital exenteration of the left eye [11,12]. In July 2012, positron emission tomography–computed tomography (PET-CT) revealed a solitary pulmonary nodule 0.5 cm in size in the right upper lobe of the patient’s lung, which had increased to 1.1 cm by September 2013 (Figure 1A). PET-CT revealed a focus of increased uptake in that nodule, with a standardized uptake value of 3.7 (Figure 1B). There was no evidence of other metastatic disease on PET-CT scans. In September 2013, the patient underwent video-assisted thoracoscopic wedge resection of the pulmonary nodule. Frozen sections using oil red O stain revealed accentuation of lipid and presences of foamy cytoplasm in tumor cells, which was positive for lipid staining (Figure 2). Permanent histology demonstrated tumor cells with foamy cytoplasm and atypical nuclei, accompanying numerous lipid globules within the cytoplasm (Figure 3), consistent with metastasis of eyelid sebaceous carcinoma. At the last follow-up, 7 months after resection, there was no loco-regional recurrence or distant metastasis of the tumor after surgery.

## Discussion

Sebaceous carcinoma of the eyelid refers to a group of carcinomas derived from sebaceous gland cells that occur in the ocular adnexa. It can be invasive in the eyelid and conjunctiva, and can metastasize to regional lymph nodes and distant organs [8,13,14]. Treatment strategies for primary eyelid sebaceous carcinoma are surgery, radiotherapy, and chemotherapy [15-17]. Distant hematogenous metastases to the lung, liver, and brain have a mortality rate as high as 30% [16,18]. However, few reports demonstrated the surgical treatment of metastatic eyelid sebaceous carcinoma.

Standard treatment strategy for pulmonary metastatic sebaceous carcinoma has not yet been established because of the limited number of cases. Chemotherapy regimens in existing reports are largely based on the combination regimens commonly used in the treatment of other forms of poorly differentiated carcinomas of the head and neck region [19,20]. Husain *et al*. reported combined chemotherapy of carboplatin and docetaxel for the patient who had multiple lung and lymph node metastases, which resulted in a 30% decrease in tumor size, but the efficacy of this regimen for sebaceous carcinoma has not yet been fully evaluated [21]. Radiotherapy for primary eyelid sebaceous carcinoma was described in several reports; however, there have been no reports describing radiotherapy for pulmonary metastatic eyelid sebaceous carcinoma [22,23]. Resection of pulmonary metastases in patients with sebaceous carcinoma is controversial. However, our case suggests that a surgical approach to lung metastasis of eyelid sebaceous carcinoma could prolong survival in certain subgroups of patients, namely, those with a limited number of metastatic nodules or a significant disease-free interval.

The possibility of metastasis from eyelid sebaceous carcinoma or primary lung cancer cannot be predicted only on the basis of radiologic findings or disease-free interval. In the present case, we could successfully differentiate solitary lung metastasis of eyelid sebaceous carcinoma from primary lung cancer using oil red O stain, which stains lipid has a red color, on frozen sections.

## Conclusion

We report a rare case of solitary lung metastasis of eyelid sebaceous carcinoma, which was successfully resected and differentiated from primary lung cancer using oil red O stain on frozen sections. Pulmonary resection is a good option for the treatment and diagnosis of metastatic eyelid sebaceous carcinoma.

## Consent

Written informed consent was obtained from the patient for the publication of this case presentation and accompanying images. A copy of the written consent is available for the review by the Editor-in-Chief of this journal.

## Abbreviations

CT: Computed tomography; FDG: Fluorodeoxyglucose; PET: Positron emission tomography.

## Competing interests

The authors declare that they have no competing interests.

## Authors’ contributions

KK and TO wrote the manuscript. KK, TO, KA, and IK performed surgery. YH and KE carried out the pathological examination. MK and TG were involved in the final editing. All authors approved the final manuscript.
